# The Critical Role of Spreading Depolarizations in Early Brain Injury: Consensus and Contention

**DOI:** 10.1007/s12028-021-01431-w

**Published:** 2022-03-07

**Authors:** R. David Andrew, Jed A. Hartings, Cenk Ayata, K. C. Brennan, Ken D. Dawson-Scully, Eszter Farkas, Oscar Herreras, Sergei. A. Kirov, Michael Müller, Nikita Ollen-Bittle, Clemens Reiffurth, Omer Revah, R. Meldrum Robertson, C. William Shuttleworth, Ghanim Ullah, Jens P. Dreier

**Affiliations:** 1 Queen’s University, Kingston, ON Canada; 2 University of Cincinnati, Cincinnati, OH USA; 3 Harvard Medical School, Harvard University, Boston, MA USA; 4 The University of Utah, Salt Lake City, UT USA; 5 Florida Atlantic University, Boca Raton, FL USA; 6 1HCEMM-USZ Cerebral Blood Flow and Metabolism Research Group, and the Department of Cell Biology and Molecular Medicine, Faculty of Science and Informatics & Faculty of Medicine, University of Szeged, Szeged, Hungary; 7 Instituto de Neurobiologia Ramon Y Cajal (Consejo Superior de Investigaciones Científicas), Madrid, Spain; 8 Medical College of Georgia, Augusta, GA USA; 9 University of Göttingen, University Medical Center Göttingen, Göttingen, Germany; 10 University of Western Ontario, London, ON Canada; 11 Center for Stroke Research Berlin, Charité – Universitätsmedizin Berlin, corporate member of Freie Universität Berlin, Humboldt-Universität zu Berlin, and Berlin Institute of Health; and the Department of Experimental Neurology, Charité – Universitätsmedizin Berlin, corporate member of Freie Universität Berlin, Humboldt-Universität zu Berlin, and Berlin Institute of Health., Berlin, Germany; 12 School of Medicine, Stanford University, Stanford, CA USA; 13 The University of New Mexico, Albuquerque, NM USA; 14 University of South Florida, Tampa, FL USA

**Keywords:** Stroke, Traumatic brain injury, Sudden cardiac arrest, Concussion, Modeling, Migraine, Ischemia, Na^+^/K^+^ pump, Huntington's disease, Alzheimer's disease, Amyotrophic lateral sclerosis, Ketamine, Penumbra, Persistent vegetative state, Dendritic beading, Brain swelling

## Abstract

**Background:**

When a patient arrives in the emergency department following a stroke, a traumatic brain injury, or sudden cardiac arrest, there is no therapeutic drug available to help protect their jeopardized neurons. One crucial reason is that we have not identified the molecular mechanisms leading to electrical failure, neuronal swelling, and blood vessel constriction in newly injured gray matter. All three result from a process termed spreading depolarization (SD). Because we only partially understand SD, we lack molecular targets and biomarkers to help neurons survive after losing their blood flow and then undergoing recurrent SD.

**Methods:**

In this review, we introduce SD as a single or recurring event, generated in gray matter following lost blood flow, which compromises the Na^+^/K^+^ pump. Electrical recovery from each SD event requires so much energy that neurons often die over minutes and hours following initial injury, independent of extracellular glutamate.

**Results:**

We discuss how SD has been investigated with various pitfalls in numerous experimental preparations, how overtaxing the Na^+^/K^+^ ATPase elicits SD. Elevated K^+^ or glutamate are unlikely natural activators of SD. We then turn to the properties of SD itself, focusing on its initiation and propagation as well as on computer modeling.

**Conclusions:**

Finally, we summarize points of consensus and contention among the authors as well as where SD research may be heading. In an accompanying review, we critique the role of the glutamate excitotoxicity theory, how it has shaped SD research, and its questionable importance to the study of early brain injury as compared with SD theory.

## Introduction: Spreading Depolarizations and Their Clinical Importance

**Fig. 1:**
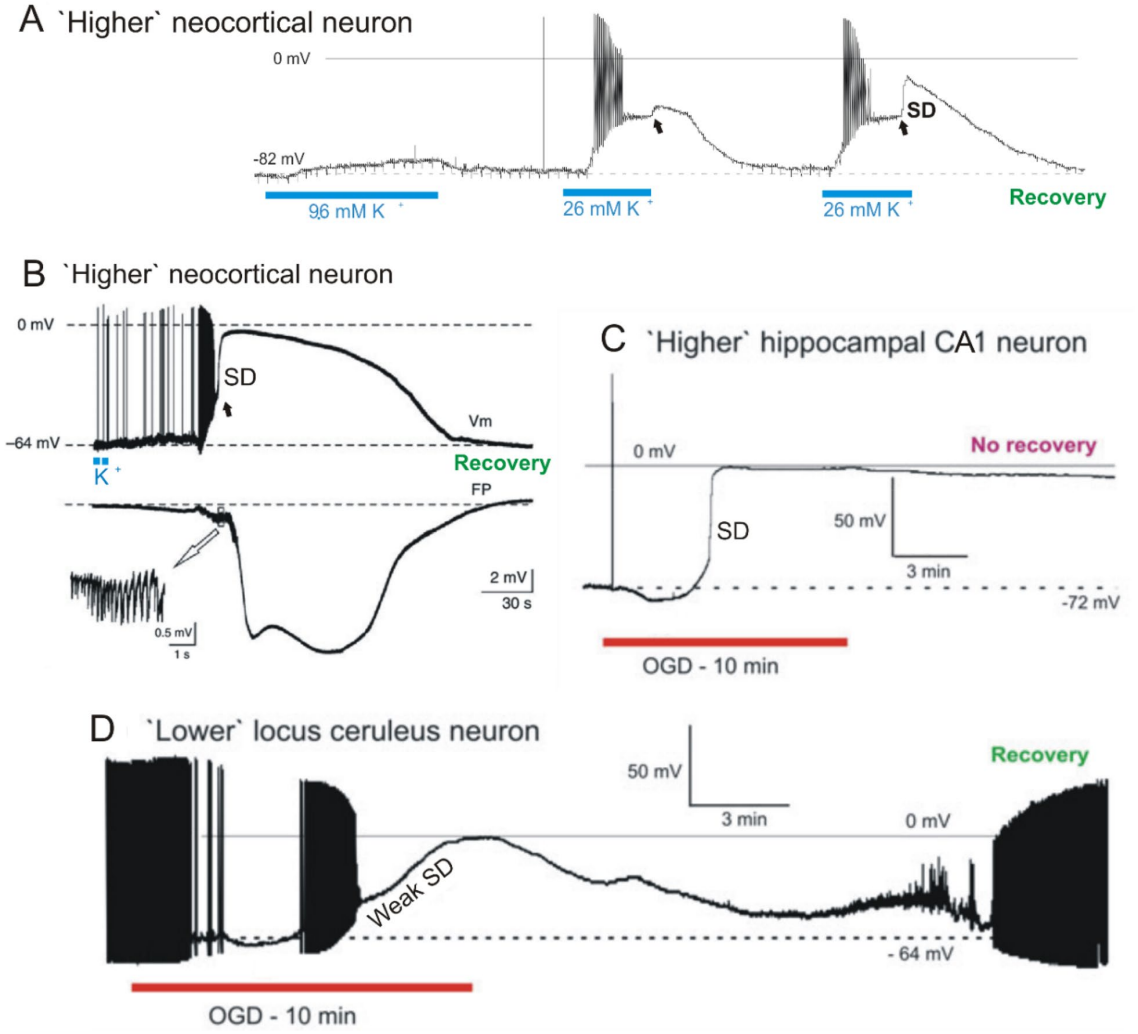
Intracellular recordings from single rodent neurons undergoing SD in live brain slices. Neurons in the higher brain briskly undergo SD while most neurons in the lower brain respond slowly and then consistently recover. **A**, In response to 5-min 9.6 mM K^+^, a rat neocortical pyramidal cell depolarizes to − 73 mV then repolarizes in control aCSF. Exposure to 26 mM K^+^ elicits more depolarization with spiking and then spike inactivation, before a plateau of − 48 mV is reached. SD onset (arrow) is aborted just as control aCSF reaches the slice. A third K^+^ exposure again evokes firing and spike inactivation, reaching a plateau at − 48 mV. A steep depolarization then coincides with SD onset (arrow). Modified from [107]. **B**, Typical membrane potential changes (Vm) of a neocortical neuron (somatosensory cortex; mouse) shown in the upper trace with a simultaneous extracellular field potential recording (FP) acquired near the recorded neuron in the lower trace. After application of KCl in layer I/II of the neocortex, neurons in layer V depolarize abruptly during CSD as the negative DC shift initiates. The inset shows a brief burst of high-frequency population spikes recorded during the early DC deflection. Modified from [228]. **C** A rat hippocampal CA1 pyramidal neuron undergoes a terminal SD induced by 10 min of OGD. Typically, higher neurons in neocortex, thalamus, striatum and hippocampus reduce their firing during OGD before undergoing rapid SD to near-zero millivolts, with no recovery. **D** A ‘lower’ locus ceruleus neuron, like most neurons in hypothalamus and brainstem, only slowly depolarizes in response to OGD. As action potentials inactivate, the membrane potential slowly continues to near-zero millivolts (Weak SD). On return to control aCSF, the neuron slowly recovers, dramatically different from higher neurons post-OGD. C and D modified from [107]

**Fig. 2:**
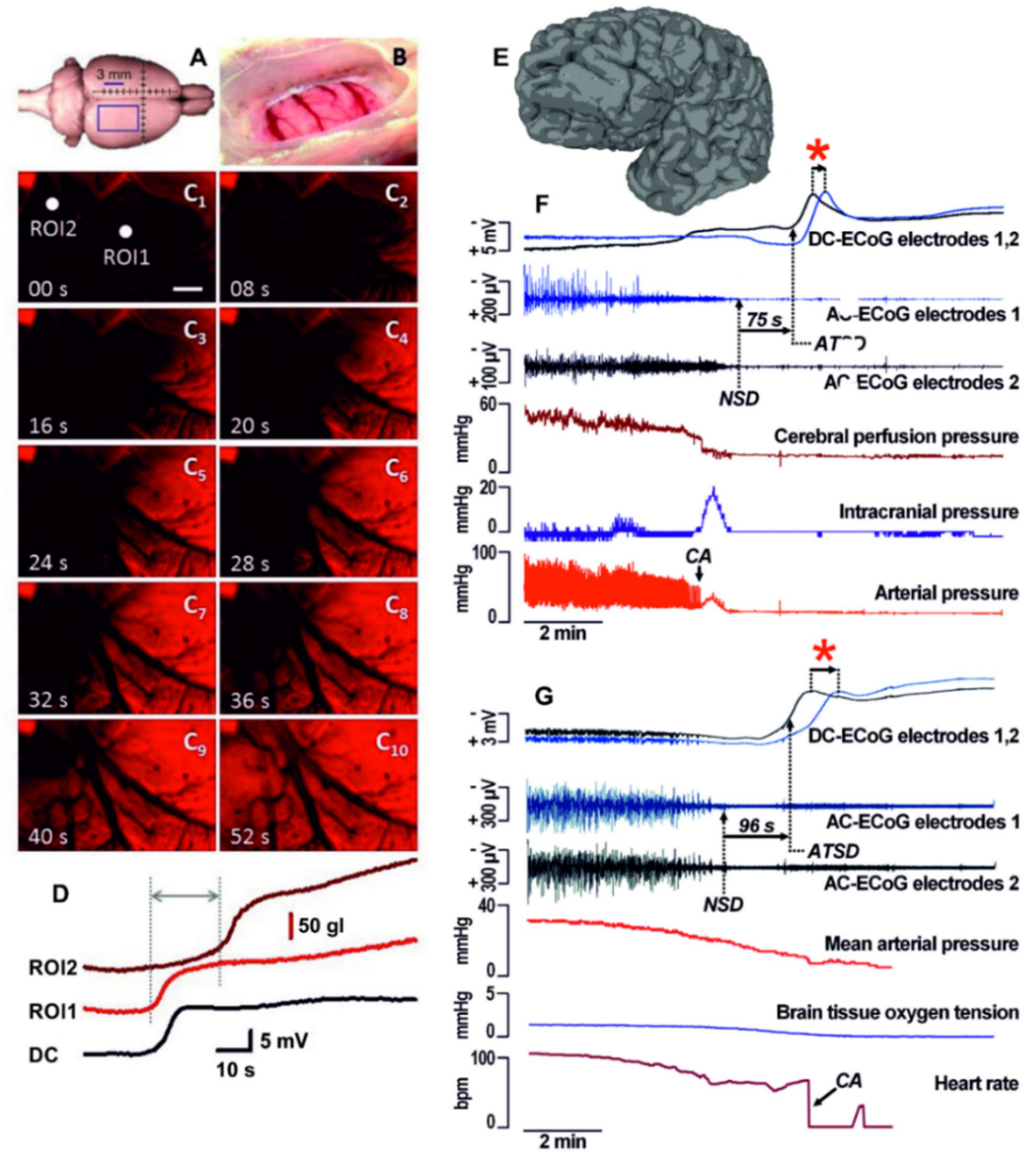
Image sequence and the kinetics of the optical signal of spreading depolarization (SD) induced by cardiac arrest in the rat cerebral cortex. **A,B** The position of the closed cranial window and the parietal cortex exposed for imaging. **C** The propagation of anoxic terminal SD as visualized by a voltage‑sensitive dye (RH1838) (Farkas et al., 2008). Note the clear wave front and the rostro‑caudal direction of propagation of the terminal SD event. The scale bar in C1 represents 500 μm. **D** The variation of the optical signal at the two regions of interest (ROIs in C 1) reveals the evolution of terminal SD. The time delay between terminal SD onset at the two ROIs confirms the propagation of the event. The rate of propagation of this particular terminal SD event was 4.1 mm/min between ROI1 and ROI2. The DC potential trace shown with inverted polarity to match the optical signal traces indicates a terminal SD acquired with electrophysiology after cardiac arrest in another rat. Note correspondence of the optical and DC potential signals (unpublished data from the Farkas laboratory). **E** Six-contact Wyler electrode strip superimposed on a geometrically discretized whole human brain taken as MPRAGE magnetic resonance imaging sequence. **F** The process of brain death in the patient begins with the simultaneous decline of arterial pressure and spontaneous cortical activity (AC-ECoG; 0.5 Hz high-pass filter, subdural electrode). Cardiac arrest then triggers a spike in intracranial pressure, likely reflecting a cerebral vasodilatory response to the sharp decrease in cerebral perfusion pressure. Within 57 s of cardiac arrest, residual synaptic activity then ceases in a non-spreading depression (NSD) of activity. After a further 75 s, terminal SD develops as evidenced by the negative shift in DC-ECoG (no filter) that spreads from electrode 2 to electrode 1. The red asterisk marks the time delay between the two electrodes suggesting the propagation of anoxic terminal SD (ATSD). The cause of death was a hepatorenal syndrome after severe aneurysmal subarachnoid hemorrhage. **G** The other patient suffered extensive shear injury and basilar skull fractures as a result of a motor vehicle accident. Electrocorticography and partial pressure of brain oxygen were monitored through a bolt placed in the right frontal lobe. Approximately three hours before death, mean arterial pressure declined to <50 mm Hg. The traces then showed a further decline in arterial pressure with a lowering heart rate as spontaneous cortical activity (AC-ECoG) decreased progressively to a state of electrical silence (NSD). Anoxic terminal SD began 96 s later. Assuming an ideal linear spread, the time delay of 41 s between negative shifts of DC-ECoG would indicate a propagation velocity of 3.2 mm/min, since intraparenchymal electrodes were separated by 2.2 mm.

At the immediate onset of brain ischemia, spreading depolarization (SD) is the principal mechanism of electrochemical membrane failure and neuronal swelling in gray matter of the higher brain [1–6]. In this review, the “higher” brain includes all structures above the hypothalamus and brainstem. Within 1–2 min of severe ischemia, depletion of the available ATP pool leads to Na^+^/K^+^ pump failure with eruption of a front of cellular depolarization that propagates at 1–9 mm/minute throughout the ischemic tissue as well as into the surrounding penumbral and normal tissue [7–11]. This SD wave drives a sudden and active jump in neuronal membrane potential to near 0 mV over seconds (Fig. 1), in contrast to most other tissues of the body that slowly depolarize over many minutes in an energy crisis. However, SD does not arise only in ischemic tissue but can also be provoked by various noxious electrical, chemical, thermal, or mechanical disturbances of gray matter. Thus, SD is associated with a range of diseases and conditions, including migraine-associated aura, concussion [12], traumatic brain injury (TBI), subarachnoid hemorrhage, intracerebral hemorrhage, ischemic stroke, circulatory arrest (Fig. 2), brain death prior to circulatory collapse, and sudden unexplained death in epilepsy [3, 13–18]. When SD invades tissue that has not already been rendered electrically silent by the triggering pathology, SD induces a loss of spontaneous and evoked activity termed “spreading depression” [19].

### Stroke and SDs

In the ischemic core, neurons will die under a maintained depolarization that typically lasts 20–30 min or more [20–26]. However, if the ischemic core is reperfused within ~ 15 min, all neurons of the ischemic core will survive, even though the neurons have been persistently depolarized for about 15 min [23, 27]. In contrast, perfusion completely ceases after cardiac arrest, and so neurons start to die after about 5 min [28]. Importantly, there are also ischemia models that do not show an ischemic core because the reduction in cerebral blood flow is milder. In such in vivo models, there is no terminal SD, but typically a cluster of recurrent moderately prolonged SDs occurs superimposed on a relatively shallow negative ultraslow potential. Yet cell death also develops [29]. A similar pattern of clustered SDs is also seen when aerobic metabolism in astrocytes is selectively blocked by aconitase inhibitors, which is associated with cell death [30, 31]. Furthermore, this pattern is recorded in the ischemic penumbra in models of middle cerebral artery occlusion or photothrombosis in numerous studies. Clustered SD events pose a particularly high metabolic challenge for recovery.

The acute injury imposed by SD has been characterized as a “continuum,” determined by the degree of metabolic stress in the tissue where SD propagates [9, 32]. This concept can be illustrated in the middle cerebral artery occlusion model in which the first SD wave persists in the core, is moderately prolonged but reversible in the penumbra, and is short lasting in the surrounding well-perfused tissue where membrane potentials recover after 1–2 min [33, 34]. The SD duration and waveform change dramatically as it propagates from the core through the penumbra and into normal tissue. There is also a continuum with respect to neuronal injury in which core neurons rapidly begin to suffer necrotic death, whereas penumbral neurons can withstand several cycles of recurrent SD and recovery before dying. In the surrounding tissue, recovery follows each SD [3, 35, 36]. Also, the pharmacological properties of the SD event change along the SD continuum, as further detailed below.

The delayed nature of penumbral SDs presents a potential therapeutic window whereby their inhibition could improve neurological outcome [37]. These SDs appear to arise as a consequence of energy supply–demand mismatch [30, 38]. The cumulative effect of many secondary SDs is a progressive deterioration of metabolic status and lesion expansion. This occurs not only because of cytotoxic membrane failure, but also because of SD evoking microvascular constriction in injured tissue with impaired neurovascular coupling. Known as “spreading ischemia” [39], this inverse hemodynamic or initially vasoconstrictive response to SD promotes prolongation of the cellular depolarization and thus cell death [3, 10, 38, 40–42]. By contrast, the normal hemodynamic response to SD in naïve, normally perfused tissue consists of a predominantly hyperemic initial phase followed by mild long-lasting oligemia after tissue repolarization [43–46]. During this normal oligemic phase, neurovascular responses to hypercapnia and functional activation are transiently disturbed, but the tissue fully recovers [47, 48]. The hemodynamic response to SD often shows a continuum across tissue from an inverse ischemic response to an increasingly normal hyperemic response [49]. Toxic factors, for example, released after subarachnoid hemorrhage, may also disrupt neurovascular coupling [3]. Tissue regions displaying inverse hemodynamic responses to SDs can display impaired autoregulation, thereby increasing the risk of irreversible tissue damage [50]. Importantly, these concepts have all been clinically translated and validated by electrocorticographic monitoring of patients with severe acute brain injury [20, 51].

### Beyond Stroke

In brain trauma as well as ischemic and hemorrhagic stroke, 50–90% of patients exhibit cortical SDs, and many show continuous, repetitive events lasting several days or even weeks after injury, with total counts of 50–100 or more. Even terminal SDs, those with no recovery, have been observed as the correlate of newly developing focal infarcts and of brain death at end of life [14, 15, 23, 52]. The full continuum from the normal hyperemic to the inverse ischemic response to SD has been found in patients with aneurysmal subarachnoid hemorrhage [23, 53, 54], TBI [50], and malignant hemispheric stroke [11].

The Na^+^/K^+^ ATPase is the main transporter regulating transmembrane cationic gradients. Its compromise leads to SD. The pump exchanges three cytosolic Na^+^ for two extracellular K^+^ via hydrolysis of adenosine triphosphate (ATP) [55]. As such, this transport is itself electrogenic, contributing several hyperpolarizing millivolts to the membrane potential. In mammalian gray matter, the Na^+^/K^+^ pump is responsible for ~ 50% of ATP hydrolysis [56]. Lack of blood oxygen and glucose inhibits ATP production, with pump failure evoking sudden SD, driven by the opening of a cryptic Na^+^/K^+^ current [57, 58]. Neurons in live slices undergo SD in response to oxygen–glucose deprivation (OGD) [59–61]. Also effective is severe hypoxia in an interface slice chamber [62, 63]. Similarly, SD is imaged and recorded in vivo under ischemia [64–66].

Despite the potential clinical relevance and the decades-long history of SD research, including recent comparative and molecular genetic approaches in insects [67–69], it is still unclear how compromise of the Na^+^/K^+^ pump links to SD generation. Essentially, we have not yet identified the molecular mechanisms underlying either the spread or the depolarization. The specific ionic channel type (or types) that opens to drive SD is not yet known, despite its hypothesized existence based on computer modeling [70] and its general characterization as a nonspecific Na^+^/K^+^ current [57, 58]. Voltage-gated Na^+^ channels also carry some inward current, and because Na^+^ influx predominates over K^+^ efflux, neurons depolarize. It is likely that the same conductance drives SD propagation through healthy, nonischemic tissues, as during migraine aura.

The purpose of this critique is to challenge and discuss several basic issues concerning the largely unknown molecular mechanisms that initiate, drive, and terminate SD. We will also highlight our issues of consensus and of contention regarding SD. Year after year, published reviews imply that the basic cellular mechanisms underlying acute neuronal death following brain ischemia are reasonably well established based on the glutamate excitotoxicity theory. In fact they are not [71]. The critical role of SD in acute brain damage has been consistently underestimated. Here we highlight areas of agreement and progress as well as those open to debate that require more research into SD. First, how failure of the Na^+^/K^+^ ATPase (the “pump”) leads to SD initiation is unclear, as well as how the SD event regenerates and propagates. Elevated levels of released extracellular K^+^ or of glutamate can help perpetuate SD propagation, but as argued below, neither appear to be a viable driver of SD within metabolically stressed gray matter. Second, the specific channel that opens to drive SD is not yet identified. Third, the processes that terminate SD and establish a period that is refractory to SD have not yet been fully determined. Recovery from SD requires, at minimum, a reactivation of the Na^+^/K^+^ ATP pump to reestablish lost ion concentration gradients. This itself will delay SD reignition [9, 72].

An important complicating aspect of SD research is that SD can be generated in isolated preparations using different forms of metabolic stress, as discussed in the next section.

## SD and the Central Role of the Na^+^/K^+^ ATPase

### What Does the Na^+^/K^+^ Pump Do?

The living animal cell has a high concentration of impermeable anions inside as well as a cell membrane permeable to K^+^ and less so to Na^+^. The pump actively removes Na^+^ and replenishes leaking K^+^. Both cations are similarly imbalanced, but K^+^ is near its electrochemical equilibrium because the negative resting potential opposes its concentration gradient. Thus, energy is associated primarily with the Na^+^ electrochemical gradient. The pump maintains these gradients and, in turn, cell volume [1].

Most treatments that initiate SD act to overwhelm or reduce the activity of the Na^+^/K^+^ ATPase. This is most obvious when using ouabain to specifically inhibit the pump, which induces SD in vivo and in slice preparations [29, 73–77]. Similarly, ischemia, OGD, or hypoxia evokes SD because the Na^+^/K^+^ pump stops working when ATP reserves are depleted. Gray matter cannot completely repolarize from SD until the ATP-dependent active transport of Na^+^ and K^+^ against their physiological gradients is reestablished. Thus, during SD, intracellular Na^+^ ([Na^+^]_i_ ) increases from 10 to ~ 40 mM, whereas intracellular K^+^ ([K^+^]_i_ ) decreases from 135 to ~ 105 mM [78]. By contrast, Na^+^ concentration outside ([Na^+^]_o_ ) decreases from 145 to 60 mM and [K^+^]_o_ increases from 3 to ~ 60 mM [79, 80]. In the presence of 3 mM ATP, the affinity for Na^+^ is about three times that of K^+^ at the cytoplasmic site of the Na^+^/K^+^ pump, whereas the affinity for K^+^ is about I00 times higher than that of Na^+^ on the extracellular site [81].

In the well-perfused neocortex, ATP levels fall by ~ 50% as nonischemic SD taxes the Na^+^/K^+^ ATPase [89]. By contrast, in the severely ischemic neocortex, ATP levels are already low, and resulting pump failure causes the SD event. The pivotal role of the Na^+^/K^+^ ATPase in clearing [K^+^]_o_ becomes apparent when its normal function is impaired. Partial pharmacological inhibition of the Na^+^/K^+^ ATPase by using a low concentration of ouabain considerably slows K^+^ removal [76, 90, 92]. Whether the recovery of Na^+^/K^+^ ATPase transport is the sole mechanism terminating SD is unknown but unlikely.

### Na^+^/K^+^ ATPase Isoforms and SD

The alpha, beta, and FXYD subunits form the Na^+^/K^+^ ATPase. Each have several isoforms, so many variants of the enzyme exist with differing properties [93]. Nevertheless, SD is a feature of insects that express only one alpha and one beta isoform and no FXYD subunit. Na^+^/K^+^ pump dysfunction also affects other linked transport processes, reducing, for example, the local extracellular/intracellular [Ca^2+^] gradient.

The alpha 1 isoform is expressed in all vertebrate cells. It has a high affinity for Na^+^ and so activates to extrude small Na^+^ increases during baseline neuronal activity, essentially serving a housekeeping function [94]. Compared with the alpha 3 isoform (below), it is much less efficient at handling Na^+^ removal during rapid discharge or during maintained depolarization. It is the most prevalent isoform in higher brain neurons, those most prone to SD, such as the neocortex, striatum, hippocampus, and thalamus [95]. So upregulating alpha 1 activity represents a target for protection or augmentation of pump function to combat ischemia. However, being expressed in every cell type, it is a less specific therapeutic target compared with alpha 2 and alpha 3 (below).

Alpha 2 is expressed in striated muscle and neural tissue, primarily astrocytes and other glia. The a2b2 complex is maximally active when [K^+^]_o_ is high and the membrane potential is depolarized and so is efficient for clearing K^+^ after intense neuronal activity. The role of the alpha 2 isoform in SD is supported by the clinical and experimental observations that mutations in *ATP1A2*, the gene encoding alpha 2, induce familial hemiplegic migraine type 2. This is a Mendelian model of disease for SD and SD-induced spreading depression of activity, resulting in the patient percept of migraine aura [96]. Reduced alpha 2 expression leads to a propensity for SD because the astrocytic alpha 2 component is less efficient [9, 92]. In addition to the model in which aerobic metabolism in astrocytes is selectively blocked by aconitase inhibitors [30], familial hemiplegic migraine type 2 provides another example that SD, although a primarily neuronal phenomenon, can also arise as a consequence of impaired astrocyte function. A third example is provided by genetically modified mice in which astrocyte-driven inactivation of connexin 43 reduces astrocytic gap junction communication, leading to increased SD susceptibility and propagation [97].

Alpha 3 is found in the heart and is highly expressed in the brain, mainly in neuronal projections [98] and dendritic spines [94]. During intense neuronal activity, the local Na^+^ concentration in dendrites and spines increases from 15 mM to as high as 100 mM [99]. The required Na^+^ clearance that follows is mainly attributed to alpha 3 [100], which has a low sodium affinity [82, 101, 102] such that alpha 1 < alpha 2 < alpha 3. The alpha-3-containing pumps in neurons appear optimized for clearance of elevated Na^+^ concentration during high-intensity neuronal firing at depolarized levels. This has implications for resilience during SD. In support, the alpha 2 and alpha 3 isoforms are proposed to reduce vulnerability to ischemia compared with alpha 1 [56, 103]. Mined data from the Allen Brain Bank [104] indicate that the hypothalamus and the brainstem neurons express a greater proportion of alpha 3 to alpha 1 compared with the higher brain regions of the neocortex, thalamus, striatum, and hippocampus [105]. This may help explain how hypothalamic neurons [106] and brainstem neurons [95] can maintain firing, better resist complete depolarization, and better recover during OGD in contrast to neurons in higher brain regions, which immediately succumb to SD. Note that the hippocampus displays an intermediate proportion of alpha 3 expression yet is susceptible to SD and ischemic damage, so an elevated neuronal alpha 3 expression does not guarantee increased resistance to ischemia [105]. The lower projection neurons resist SD evoked by OGD or ouabain [107] or by high [K^+^]_o_ [108], likely because alpha 3 more efficiently removes higher levels of intracellular Na^+^. Therefore, compared with the alpha 1 isoform, alpha 3 can, in theory, better handle large Na^+^ loads in electrically active cells and, by extension, during SD. Increasing alpha 3 expression in neurons might improve postischemia resilience and survival.

### Elevating [K^+^]_o_ and Pump Inhibition

Raising [K^+^]_o_ is a common technique to induce SD (see Modes of SD induction), but how it activates SD is still unclear. At the level of the single cortical neuron, SD induced by raising [K^+^]_o_ (Fig. 1A, B) is different from SD induced by OGD (Fig. 1C). From the Nernst equation for K^+^, high [K^+^]_o_ directly depolarizes neurons, thereby activating voltage-sensitive Na^+^ channels and so promoting discharge. With further depolarization, action potentials inactivate. Graded K^+^ application (Fig. 1A) indicates that this passive depolarization is separate and distinct from the SD current in higher brain neurons (Fig. 1A, arrows). Presumably the Na^+^/K^+^ ATPase is compromised, activating the SD current, but this requires further research.

In contrast, hypoxia or OGD initially quiets firing by dampening synaptic input and briefly hyperpolarizing the neuron before SD current is activated (Fig. 1C) [95, 109]. In higher brain slices, the one-two punch of metabolic stress and SD means that the neuron has trouble recovering. In contrast (Fig. 1D), neurons from hypothalamus [106] and brainstem [95] undergo weak SD and more easily repolarize.

The normally functioning pump clears [Na^+^]_i_ and [K^+^]_o_, both of which increase during neuronal firing. The pump also compensates for cell swelling. It essentially protects against SD onset. And yet the glutamate excitotoxicity theory ignores the Na^+^/K^+^ pump as a major player in the development of acute brain injury [71].

## Variability in Experimental Preparations Used for Investigating SD

There are several variables which have complicated research aiming to reach a consensus on the mechanisms evoking and driving SD. See also a discussion of `false positives` that can mistakenly imply SD is blocked [71].

### Terminology

One source of confusion in the literature is equating the terms ‘ischemia,’ ‘oxygen–glucose deprivation’ and ‘hypoxia.’ Although hypoxia is an important component of all three conditions, the three terms should be used precisely. ‘Ischemia’ should be reserved for in vivo models characterized by (1) lack of oxygen, (2) lack of glucose, and (3) lack of perfusion. Hypoxic-ischemic brain injury [110] is related to a frequent scenario in the clinic when the patient is deeply anesthetized and stops breathing, which requires intubation. Otherwise, the patient becomes hypoxic, affecting cerebral and cardiovascular function [15]. The hypoxia can evoke cardiac arrest, causing a double hit to the brain from initial hypoxia and subsequent global ischemia. Only models that precisely reproduce this clinical scenario should be called models of hypoxic-ischemic brain injury.

In addition, the term ‘peri-infarct depolarization’ (PID) is misleading and should be avoided [20, 32]. It is better to use the term ‘spreading depolarization’ and to refer to the SD continuum. PID implies being able to precisely differentiate ischemic regions of the core, penumbra, and surrounding healthy tissue. But ischemic lesion development follows a dynamic continuum in space and time. This has been argued in the aforementioned articles and will not be detailed here [38]. The term ‘PID’ supports the incorrect concept that SDs are the consequence of the infarct. In fact, SDs precede the development of the infarct and initiate the cascades leading to the infarct.

Another source of confusion is that SD may be accompanied by two different depression patterns. It can be preceded by nonspreading depression in severely energy-compromised tissue, or it can cause a spreading depression of activity in less ischemic or well-supplied tissue. If the first SD begins in the core area of focal ischemia, after a nonspreading depression of spontaneous activity has developed there about 2 min before, the SD can no longer induce a spreading depression of spontaneous activity there because there is no activity to depress. However, when the SD wave propagates through the penumbra, where spontaneous activity is still present, it leads to a spreading depression of that activity. In clinical recordings, it is important that spreading depression of activity is defined because SDs in electrically silent tissue are associated with worse patient outcome than SDs inducing depression of activity that spreads [111].

Also confusing, equating ischemia with hypoxia in the slice literature [112] is misleading because hypoxia-induced SD is not equivalent to OGD-induced SD. With hypoxia, there can be more robust recovery, and therefore putative neuroprotectants can appear more potent. Additionally, blocking glutamate receptors can inhibit the onset of hypoxic SD, whereas OGD usually only delays SD [71] (see Extracellular K^+^ promotes SD but is unlikely to be the natural SD activator).

### Modes of SD Induction

There are several experimental slice techniques to induce SD, each varying considerably in their level of metabolic stress that compromises the Na^+^/K^+^ pump. These stressors include (in reducing order of metabolic load) the following: chemical ischemia (metabolic inhibitors combined with OGD) [113, 114], blockade of mitochondrial respiration [115], OGD alone [59, 60, 88, 116], exposure to the pump inhibitor ouabain [73, 75], hyperthermia [117], and hypoxia in interface slices (i.e., oxygen removed with reduced glucose) [7, 118]. Also, severe hypoglycemia induces SD in vivo [119]. Finally veratridine, which blocks Na^+^ channel closure (inactivation), causing abnormally high and sustained Na^+^ influx, is another inducer of SD [120].

There are less metabolically demanding techniques to evoke SD in vivo, usually involving raising [K^+^]_o_. These include prolonged elevation of [K^+^]_o_ at mildly increased concentrations [121–123] and prolonged elevation of [K^+^]_o_ at higher concentrations [124, 125]. Additionally, SD can be evoked focally by local K^+^ ejection in vivo [13, 123, 126, 127] or in slices [128]. Also in slices, brief elevation of bath [K^+^]_o_ allows induction of SD (Fig. 1A, B) repeatedly with recovery [88, 129, 130]. It is also important to note that raising [K^+^]_o_ for a sufficiently long time will produce a terminal SD [131]. This eventually results in neuronal death but takes longer than does ischemia [72, 132]. Focal induction of SD in vivo is also possible optogenetically [47], by using pin prick [133], or by using strong electrical stimulation [23, 134]. The latter is far more intense than even the most pathological brain activity and is associated with histopathological evidence of a small lesion [23].

Highly unphysiologically hypoosmotic media can promote SD in slices [135]. This is likely the result of greatly elevated excitability and synchronicity involving combined increases in the strength of synaptic transmission [136], elevated field effects [137], and intrinsic neuronal excitability [138], as reviewed in [1]. An acute decrease in plasma osmolality in intact mammals will quickly elicit seizure activity [139, 140]. However, SD associated with dilute plasma osmolality, even at pathophysiological levels, has not been reported in patients.

We conclude that SD is a highly conserved response by the brain’s gray matter that can be evoked by a plethora of stressors. And the properties of SD varies as it propagates further away from the site of action of the stressor [9, 32]. This continuum complicates research on SD and on neuroprotection in general.

### Experimental Temperature

The metabolic stressors used to induce SD have been tested in vitro at different temperatures ranging from 28 to 37 °C. Higher temperature imposes a greater metabolic challenge, leading to earlier failure of the Na^+^/K^+^ pump and thus earlier SD onset. In rodent brain slices, temperatures > 40 °C promote SD onset [141]. However, similar to the condition of high [K^+^]_O_ [76], previous data in the immature hippocampus suggest that this is not necessarily due to a lack of ATP [142] but rather overwhelming of the working pump’s transport capacity. On the other hand, lower temperature within the 28–37 °C range is neuroprotective partly because it reduces metabolic demand for ATP, thereby lowering propensity for SD [46, 60, 143]. So a drug or other treatment suspected of inhibiting SD onset, say at 32 °C, will appear more effective than at 37 °C. Therefore, it is more physiologically relevant to test potential SD inhibitors near body temperature, particularly with respect to simulated ischemia.

Regarding in vivo studies, TBI patients with hourly core temperatures of > 38.4 °C have a 63% chance of depolarization, compared with only 21% in patients with temperatures of ≤ 38.4 °C [144]. This is consistent with the finding that induced hyperthermia increases SD frequency following focal cerebral ischemia in the rat [143]. Higher temperature pushes the balance of energy toward deficit. Additionally, hyperthermia increased SD frequency in the comatose patient, and SD events themselves increased brain temperature but not body temperature [145]. Local brain temperature was also elevated during subarachnoid hemorrhage in patients who showed a terminal SD cluster, whereas delayed cerebral infarction developed at the recording site [23]. Notably, SD itself will increase brain temperature: SD releases ~ 90% of Gibbs free energy (i.e., the energy associated with a chemical reaction that can be used to do work), which is normally contained in the ion concentration gradients across the neuronal membranes. This energy is then converted to heat [125].

### Age of the Experimental Animal

A second likely source of variability is that investigations in rodents in vivo and in brain slices have been performed across a substantial age range. Advancing age and ischemia appear to elevate the thresholds of SD both in vivo [146] and in brain slices [147, 148]. On the other hand, neonatal brains do not seem to support SD [149]. However, OGD-induced SD is already found by P5 in rats [150]. Milder SD stimuli may fail until postnatal day 10 and continue to fail occasionally up until day 20 [151]. However, SD susceptibility of the tissue then rapidly reaches its maximum at 16–30 days of age and slowly decreases again thereafter [147, 148, 152]. So in general, age correlates negatively with SD propagation [153].

### The CNS Region Under Study

A third variation is that SD has been studied using slices from several brain regions, including the neocortex, hippocampus, striatum, thalamus/hypothalamus, brainstem, and cerebellum, in addition to the excised retina. First, the mouse primary sensory cortex is more susceptible to SD than other neocortical regions exposed to anoxia, a trend also seen in patients with TBI [8].

Next, gray matter in the hypothalamus and the brainstem are significantly more resistant to OGD-evoked SD than in higher brain regions [107, 154] as shown in Fig. 1D. In fact, it is not possible to elicit full-blown SD using high K^+^ artificial cerebrospinal fluid (aCSF) in slices of the hypothalamus or brainstem [108, 155]. The reason is still unclear, but this SD resistance helps explain why the hypothalamus and brainstem suffer less damage following global ischemia, possibly promoting the emergence of the persistent vegetative state.

As another example of regional susceptibility, AMPAR activation of Purkinje cells in cerebellar slices appear to have a greater role in SD generation [155] than in other brain regions. Severe ischemia is a potent stimulus of SD in the cerebellum [156–158]. However, selective neuronal necrosis of the highly vulnerable Purkinje cells could also result from decreases in blood flow and tissue partial pressure of oxygen when ischemia was too mild to cause an SD [159]. Under identical conditions in the neocortex, selective neuronal necrosis was only observed in the hypoperfused cortex when either the mild ischemia triggered at least one SD or an SD was chemically triggered at a distance and invaded the hypoperfused zone [29, 160]. This means that Purkinje cells may have the lowest ischemic threshold for cell death in the CNS.

Regarding spinal cord gray matter, George Somjen wrote that it was “immune” to SD ([63], p. 260) but that under “special conditions,” SD could be evoked in a minority of slices [162]. However, that study used infant mice under hypoxia, and so SD would be more difficult to elicit compared with adult slices of the spinal cord using OGD (adult slices of spinal cord are exceedingly difficult to keep viable). It was later shown that the dorsal and ventral horns clearly undergo SD in response to ischemia both in vivo [163] and in response to OGD using spinal cord slices [164]. It has been suggested that, like higher gray matter, this SD susceptibility reflects greater vulnerability of the spinal cord to contusion injury (similar to higher brain) as compared with the better protected brainstem [107].

### Submerged Versus Interfaced Brain Slices

Compared with being submerged, slices held at a gas–liquid interface display a higher baseline excitability for several reasons. They acquire a compacted extracellular space caused by tissue flattening imposed by high surface tension at the gas/fluid boundary [165]. Thus, ephaptic interactions (field effects) are increased, promoting local synchronous discharge among neuronal populations particularly in laminated cortical gray matter [166]. Whether field effects can promote SD onset is debatable. However, released glutamate and K^+^ levels are more concentrated within the compacted tissue, further increasing electrophysiological excitability compared with submerged slices. Glucose is more slowly replenished because the slice is only perfused from below, so these slices are likely somewhat hypoglycemic when made anoxic. Anoxic interface slices show good recovery from SD when the slices are reoxygenated after a couple of minutes. This contrasts with OGD in submerged slices, which is more a model for global ischemia after cardiac arrest without resuscitation attempts.

Two disadvantages to interface slices are the slow diffusion of drugs into and out of the preparation and the difficulty in acquiring imaging data when objective lens submersion is required, as with multiphoton laser microscopy [2, 167]. George Somjen’s laboratory commonly used interface slices combined with severe hypoxia to induce SD [168, 169]. The interface preparation allowed rapid restoration of oxygen, leading to near-complete recovery of slice health and enabling the induction of repeated SD events in a single slice. Hypoxia alone is not sufficient to induce SD in submerged slices [170]. Slices that are made hypoxic are used to simulate clinical situations that produce hypoxemia, such as apnea, asphyxia, respiratory failure (i.e., acute respiratory distress syndrome), drowning, and high altitude, showing similar modifications of SD as observed under hypoxia in vivo [15], whereas slices deprived of oxygen and glucose (OGD) simulate the condition of severe ischemia.

In both hypoxic interface slices and submerged slices exposed to OGD, normal action potential-evoked synaptic release fails a minute or more prior to SD onset [161]. In in vivo models of ischemia, hypoxic interface slices and OGD paradigms in submerged slices demonstrate that synaptic failure occurs rapidly and precedes anoxic SD [95, 118, 171, 172]. In the case of hypoxia, failure of synaptic transmission can persist over hours [173]. The evoked field excitatory postsynaptic potential is lost about 2 min before SD onset in neocortical slices exposed to OGD [59, 174, 175]. This early electrical inactivity that precedes SD in severe energy compromise is associated with a hyperpolarization of neurons both in slices and in vivo [61, 80, 176]. Spontaneous release of small quanta of neurotransmitters, such as gamma minobutryic acid (GABA) and glutamate, is also recorded [118]. Also, adenosine release may inhibit synaptic transmission even ahead of the transmitter pool of glutamate becoming exhausted [115].

Kunkler and Kraig [177] found that SD could be initiated only rarely in organotypic slices submerged under normal saline superfusion. Acute slice models of hypoxia, and particularly of OGD, simulate severe energy deficiency, whereas brief K^+^ elevation simulates milder stress. In all cases, the result are dramatic SD events that are easily recorded or imaged. Slices are also advantageous for investigating whether an SD-related mechanism is still present in the absence of intact blood circulation.

## The Channel Type(s) Initiating and Driving SD

We have yet to pinpoint a major source of inward current driving SD. The main conductance driving SD is known to be a dual Na^+^ influx/K^+^ efflux [58], but the actual channel that opens is not yet identified. Computer models often rely on a persistent inward voltage-sensitive Na^+^ current (see Issues of Contention Among the Authors Regarding SD), but these channels are of low density on neurons and drive only minor depolarization. SD proceeds in the presence of voltage-sensitive Na^+^ channel blockers [71]. Müller and Somjen [169] showed that when tetrodotoxin (TTX, which blocks action potentials) was added to selective n-methyl-d-aspartate (NMDA) and non-NMDA glutamate antagonists, SD was prevented in half the hypoxic slices. When SD did occur, it was delayed, yet neurons depolarized to the same extent as in normal solution. The SD-related sudden decrease in [Na^+^]_o_ was depressed by only 19% in the presence of all three drugs, indicating that Na^+^ flows into neurons other than through ionotropic glutamate receptors and TTX-sensitive Na^+^ channels, presumably through an, as yet, unidentified nonspecific Na^+^/K^+^ channel [58].

Given the lack of viable candidates, it is probable that the initial SD and the ensuing penumbral SDs are driven by the opening of a still unidentified channel [178, 179] that is normally inactive in healthy, nonischemic gray matter [178]. It could become modified from an existing channel that then can conduct larger molecules under ischemic conditions, a so-called megachannel, as discussed by Anderson et al. [59]. Fujiwara et al. in 1992 [180] originally showed that carboxyfluorescein (MW 376 Da) leaks rapidly from blebbing CA1 neurons during 5 to 7 min of OGD, leading Tanaka et al. [181] in 1999 to predict “the formation of micro-pores in the small blebs may generate the rapid depolarization (i.e., SD) because it is voltage-independent and is due to a nonselective increase in permeability to all participating ions, which probably occurs only in pathological conditions.” Calcein (MW 623 Da) and SR-101 (MW 607 Da) also leak on exposure to OGD [182]. Pannexin 1 had been a megachannel candidate because it opens after SD onset, providing a further conduit for the flux of ions during SD [182]. Nevertheless, OGD-induced SD still proceeds and causes acute neuronal damage in the presence of the pannexin 1 antagonist carbenoxolone [174]. In vivo, neither SDs in normal tissue nor SDs induced by moderate ischemia are inhibited by carbenoxolone [183, 184]. Moreover probenecid, another pannexin 1 antagonist, does not inhibit hypoxia-induced SD [118] or OGD-induced SD [185]. Typically, the story is less clear for K^+^-evoked SD, in which selective pharmacological inhibition of the P2X7 channel does not affect SD but inhibiting of the P2X7–PANX1 pore complex reduces SD frequency and duration [186].

Alternately, an open SD channel might arise as a conversion from what is normally a transporter molecule. Membrane patch- clamp studies provide elegant evidence that the marine poison palytoxin transforms the Na^+^/K^+^ ATPase into an open channel in cardiac myocytes and human embryonic kidney  (HEK) 293 cells [187, 188], mammalian erythrocytes [189], and frog oocytes [190]. In a similar fashion, maitotoxin binds to the plasma membrane Ca^2+^ ATPase and turns it into a Ca^2+^-permeable ion channel [191]. Conversion from pump to channel is not surprising given the evolutionary link between transporters and channels, in which mutations can convert one to the other [192–194]. Perhaps metabolic stress can do the same. Recent evidence indicates that an SD-like inward current is generated by the Na^+^/K^+^-ATPase conversion from transporter to channel in the presence of palytoxin. Andrew et al. [195] proposed that this toxin might mimic a released molecule that binds to and opens the pump, generating SD. Compromise of the Na^+^/K^+^ pump clearly initiates SD, but whether ischemia can convert it to an open channel is an important question.

## Extracellular K^+^ Promotes SD But is Unlikely to be the Natural SD Activator

The mechanism of SD propagation is controversial, although it apparently follows a nonlinear reaction/diffusion process [196–199]. That is, neurons (or possibly other brain cells) release a neuroactive substance that diffuses to adjacent cells, where it triggers in neurons a depolarizing, self-propagating, regenerative process. Essentially, SD is induced at every spot in the tissue reached by the SD wave. Among candidates for a humoral factor, K^+^ and glutamate are commonly considered. Glutamate was discussed as unlikely [71]. Regarding K^+^, there are also significant arguments against it being the key mediator of SD propagation.

First, at a given point in gray matter, [K^+^]_o_ does not begin to significantly increase before the negative DC shift is underway [79, 115, 121, 125]. Yet the concept is so influential that the steep rise in [K^+^]_o_ can be found illustrated ahead of the negative DC shift [200]. Note that under severe hypoxia or ischemia, or if the experimenter applies K^+^, a minor increase in [K^+^]_o_ arises prior to SD (Fig. 1B).

Second, the magnitude of [K^+^]_o_ necessary for SD ignition casts doubt that diffusion of [K^+^]_o_ is sufficiently high enough to sequentially drive SD initiation as it propagates. At the SD front, where neuronal depolarization is just under way, measured [K^+^]_o_ is barely above baseline, so it cannot be continually reinitiating SD as the front propagates. The same rationale holds for glutamate as an SD initiator.

Third, it is difficult to explain how tissue with a maintained and elevated [K^+^]_o_ of ~ 15 mM (the approximate minimum to evoke SD) can maintain well-configured and regular SD events, as observed both in vivo and in brain slices.

Why does flooding the slice with physiological levels of bath-applied glutamate (0.1–1.0 mM) or of NMDA or kainate fail to induce an organized spreading event, whereas elevating [K^+^]_o_ induces spread? Both released K^+^ and released glutamate will initially be buffered by glial reuptake mechanisms that will be quickly swamped, leading to depolarization of the entire field of neurons. Yet an SD is evoked by bath-applied K^+^ but not bath-applied glutamate. One explanation is that elevated [K^+^]_o_ directly decreases pump activity, whereas massive glutamate receptor activation simply depolarizes every neuron at once, later leading to pump overload. We have hypothesized that pump compromise is a prerequisite for generating a propagating event (see SD and the central role of the Na  ^+^/K ^+^ ATPase). Clearly, the way to fully refute the K^+^ hypothesis is to chemically identify a factor that is released by gray matter in response to metabolic stress which then becomes elevated just prior to SD onset.

There is a consensus that released K^+^ helps drive SD propagation*.* Hypoxia- or ischemia-evoked SD is preceded by a slow increase in [K^+^]_o_ [29, 80, 109]. This may contribute to the ignition of SD in the energy-deficient brain region because, as shown by the Nernst equation for K^+^, high [K^+^]_o_ directly depolarizes neurons and astrocytes. In neurons, this will open V-sensitive Na^+^ channels [201] as well as a small persistent neuronal Na^+^ conductance [202]. Also contributing to the depolarization, high [K^+^]_o_ reduces glutamate uptake by the excitatory amino acid transporters [203, 204]. But the main driver of SD is the Na^+^/K^+^ conductance [58] through an, as yet, unidentified channel type.

## Hints of a Released Activator of SD that is Neither Glutamate Nor K^+^

The slow migration of SD suggests that a chemical activator could be released by stressed gray matter extracellularly just ahead of the SD front. The initiator has often been assumed to be released K^+^ [205], but as noted with glutamate, there is the cause/effect issue as well as other problems [71]. One hypothesis is that an as yet unidentified SD activator (SDa) may be released by gray matter, acting to inhibit the pump. That an SDa might both initiate and promote SD regeneration is an intriguing idea. Martins-Ferreira et al. [206] found that the medium bathing isolated retinas undergoing light-evoked SD could trigger SD in another otherwise untreated retina. Moreover, Kunkler and Kraig [177] found that SD could be initiated only rarely in organotypic slices submerged under normal saline superfusion. However, in an interface chamber where saline exposure was restricted to the bottom surface of the cultures, SD could be initiated by electrical stimulation. They suggested that a soluble substance released to the interstitial space was essential for SD. Pomper et al. [207], also using hippocampal slice cultures, found that repetitive SD-like events at intervals of 10–15 min increased in duration and decreased in threshold for induction by electrical stimulation. The propensity for SD, however, does not increase after daily SD induction for 1–2 weeks [208], so there is no apparent cumulative effect over hours and days. Theoretically, an ideal SDa might potently inhibit the pump but quickly inactivate once the metabolic stress was removed. As several research groups have hypothesized an SDa, perhaps it reflects a dissatisfaction with K^+^ or glutamate fulfilling this role.

An alternate scenario is that an intracellularly transmitted SDa might pass through gap junctions between astrocytes, which are tightly electrically coupled, and adult neurons likewise might display enough coupling to support a similar spread among neurons. For instance, the positive field spikes that herald SD in the hippocampus were interpreted as return currents for negative spikes in adjacent neurons using a transcellular path for current [209]. However, blockers of gap junctions are not specific and have inconsistent effects on SD (p. 312–3 in 268). For example, neither carbenoxolone nor probenecid delayed or blocked OGD–SD in brain slices [118, 183, 210]. SDs induced by moderate ischemia in vivo were not inhibited by carbenoxolone either. Octanol and other long-chain alcohols inhibit gap junctions and SDs [211], but they are not specific [184]. For example, octanol blocks voltage-dependent sodium currents [212], potentiates GABA_A_ receptors [213], and inhibits N-methly-d-aspartate receptors (NMDARs ) [213, 214]. Nevertheless, it remains a possibility that a small (< 1,000 Da) signaling molecule could move through open connexons of the astrocytic syncytium to activate and/or promote propagation of SD. Mouse models knocking out connexons have proven inconclusive because of the possibility of developmental compensation.

## Computer Modeling of SD

The first computational model for SD was proposed by Alan Hodgkin and published in a 1963 article by Bernice Grafstein [205]. For the 4 decades that followed, computational models were more concerned with the propagation of SD than its initiation and the biophysical mechanism behind it. These models mostly followed three different theories about SD propagation: (1) K^+^ diffusion through extracellular space [196], (2) glutamate release from intracellular stores and diffusion through extracellular space [215], and (3) diffusion of K^+^ through gap junctions between neurons [216]. The model by Hodgkin and Grafstein was based on the K^+^ diffusion hypothesis [205], in which the governing equations included K^+^ diffusion and two phenomenological terms representing the release of K^+^ from neuronal intracellular space and recovery (clearance) by unspecified pathways. A study in 1978 [218] generalized this formalism to allow for Na^+^, Cl^−^, K^+^, and Ca^2+^ concentrations in multiple compartments.

Although most of these models give a reasonable estimate for the velocity and other basic features of SD waves, they present a simplified macroscopic view of several complex underlying microscopic mechanisms. These models were mostly inspired by the similarities between the neuronal action potential and the SD wave. Both are considered all-or-none events, in which the trajectory takes its due course controlled by inward and outward currents that are activated over different time scales. Thus, like action potential, an SD event can be represented by a simple FitzHugh–Nagumo-like formalism. That is,

1 $$\frac{\partial C}{\partial t}=D\frac{{\partial }^{2}C}{\partial {x}^{2}}+F\left(C,R\right)+{I}_{stim}$$

and

2 $$\frac{\partial R}{\partial t}=G\left(C,R\right),$$

where *C* and *D* represent the concentration and diffusion coefficient of K^+^ and glutamate, respectively. The nonlinear term *F*(*C*,*R*) models the release and recovery (uptake) of K^+^ or glutamate, and *R* is the recovery variable with linear dependence on *C* and *R* (*G*(*C*,*R*)). *I*_stim_ is the stimulus igniting the SD wave, such as a local increase in K^+^ or glutamate level. However, the actual mechanism causing this stimulus is never specified. Furthermore, the release and recovery terms are often phenomenological in nature and might not necessarily represent the underlying biophysical processes.

More sophisticated models have evolved over the past 20 years but make several assumptions that may or may not be correct. First, most SD models generally assume that a persistent voltage-dependent Na^+^ conductance is the primary conductance driving SD. Being voltage-sensitive, they should start inactivating at ~  − 30 mV, so it is unclear how neurons are driven to 0 mV during SD. Alternately in Somjen’s laboratory, Czeh et al. [57] used TTX and other blockers to demonstrate a nonspecific Na^+^/K^+^ conductance driving the neurons to 0 mV during SD. However, a channel candidate was lacking at that time, so the Somjen laboratory turned to modeling an activated persistent Na^+^ current and an NMDA-controlled current [219]. In pyramidal neurons, persistent Na^+^ channels exist at low density on cell bodies and dendrites (compared with the axonal initial segment) [176], making those channels an unlikely source of the formidable SD current. Elevating [K^+^]_o_ could enhance the persistent Na^+^ current [202], so it probably supports SD. Less conventional scenarios, such as the opening of a megachannel or conversion of a transporter to a channel, have not yet been modeled. In a model mimicking OGD conditions, neuronal membrane potential reaches 0 mV when the Na^+^/K^+^ pump is completely blocked [220]. However, for that scenario, ATP levels would have to be near 0, which might not be reached in the tissue. Modeling how an SD event terminates has also been challenging. Presumably with return of oxygen and glucose, the initially open channels close. Also, K^+^ uptake by glia and return of pump function aid in repolarization. Models may also include activation of a K^+^ current analogous to the K^+^ rectifier, although there is little evidence for such a current from neuronal recordings. Release of adenosine or other, as yet, unidentified messengers that inhibit SD may occur.

## Issues of Consensus Regarding SD Among the Authors

SD evoked by ischemia of focal stroke, cardiac arrest, subarachnoid hemorrhage, or TBI is itself a major contributor to the neuronal injury that follows. But the preeminence of glutamate excitotoxicity is continually reinforced by new review articles particularly on stroke that do not question the excitotoxicity theory, do not provide new experimental support, and do not consider the important role of SD in ischemic brain damage as reviewed in [71]. Meanwhile, recent reviews on other neurodegenerative diseases (Alzheimer`s disease, Huntington`s disease, amyotrophic lateral sclerosis, traumatic brain injury (AD, HD, ALS, TBI) have abandoned glutamate excitotoxicity as causal [71]. We concur that more data are required to further support causality between SD and injury and certainly between glutamate release and long-term brain injury.

- Experimental variables when using acute brain slices to study SD can confound results. OGD-evoked SD is not equivalent to severe hypoxia, which is less metabolically demanding. We argue for more standardized parameters when testing ischemia-like stress in brain slices.
- The molecular mechanisms leading to SD initiation are still unclear. Moreover, how SD regeneration drives propagation is poorly understood. Elevated levels of released extracellular K^+^ or of glutamate can help promote SD propagation, but neither is a credible candidate as the biological initiator/perpetuator of SD. Both do play a role in K^+^-evoked and optogenetically evoked SD. Alternately, there are hints that one or more small molecules may be released from gray matter during ischemia to initiate and then promote SD regeneration.
- The channel type (or types) that then opens to drive SD is still unidentified but is known to conduct both Na^+^ and K^+^, which explains why neurons depolarize to near 0 mV. It would be useful to modify current computer models of SD in this regard because TTX studies show that opening of the voltage-dependent Na^+^ channel initially helps drive depolarization but then it inactivates between − 30 and − 20 mV.
- To make meaningful predictions and help resolve some of these issues, computer models must transition from the phenomenological approach, in which the focus is usually on reproducing a single experiment, to a biophysical paradigm, in which the model can explain all observations about SD. The formalism of key factors related to SD, such as the kinetics of the Na^+^/K^+^ pump, glutamate homeostasis, the spatiotemporal dynamics of extracellular K^+^, and the related pathways, has to be based on biophysical facts. Furthermore, the glial network, vasculature, inhibitory networks, and GABA homeostasis should be essential components of SD models because they all have roles in SD.
- Susceptibility to SD varies among brain regions. For example hypothalamic and brainstem nuclei of the adult rodent do not support strong SD [95, 107] unless chemically depolarized [221] or preconditioned with high K^+^ [222]. This SD resistance can help account for hypothalamic and brainstem survival post ischemia in patients in a persistent vegetative state. The increased susceptibility of the immature brainstem to SD has been advanced as a possible explanation for sudden unexplained death in epilepsy [13, 16].
- There is also variability in SD susceptibility across neocortical gray matter [8, 197, 223, 224]. Again, SD is not simply a default of mammalian gray matter. Differences in SD susceptibility can be clinically relevant. One underlying reason may be differential expression of Na^+^/K^+^ pump isoforms across brain regions as well as across their constituent neuronal populations.

## Issues of Contention Among the Authors Regarding SD

- How does elevated [K^+^]_o_ generate experimental SD? Elevated [K^+^]_o_ activates the pump at 5–8 mM, at which point pump efficiency peaks. However, once it approaches 15 mM, SD is evoked, which implicates suppression of pump activity at that point. How Na^+^/K^+^ pump transport declines in the presence of elevated [K^+^]_o_ is an important issue that requires further experimentation to clarify.
- There is indirect evidence that an endogenously generated activator of SD, neither glutamate nor K^+^, is released by metabolically stressed brain tissue. Experiments can be devised to test this idea. The specific molecular mechanisms underlying SD initiation, propagation, and arrest have not yet been identified. Computer modeling of the release and diffusion of a theoretical SDa and the resulting opening of nonspecific Na^+^/K^+^ channels are interesting questions for future study. Brain metabolic stress leading to release of a molecule that initiates cell depolarization could represent the long sought-after local “oxygen detector” that represents a sentinel of hypoxic threat, as suggested by Somjen in 2004 (p. 328 in [161]).
- We know that processes are activated over seconds and minutes to terminate SD and establish a period refractory to renewed SD. It is unclear if the kinetics of pump reactivation alone can fully account for SD termination and the refractory period, which can last hours. Alternately, SD itself might lead to the release of one or more chemical messengers that inhibit the reonset of SD over minutes and possibly hours. For example, adenosine is a glutamatergic synaptic inhibitor, and its release is correlated with SD. As SD duration increases, more adenosine is released [225].
- It is unclear if SD initiation, propagation, and duration each represent separate phenomena driven by distinct molecular and electrophysiological mechanisms. The simplest scenario is that all three aspects are essentially versions of a single process whereby depolarizing channels open and stay open until the Na^+^/K^+^ pump can at least partly recover. Some authors believe that the process is more complicated.
- Reperfusion injury is a potential mechanism for lesion expansion, although the pathophysiology is unclear. Kloner et al. [226] recently reviewed the no-reflow phenomenon, suggesting that poor reperfusion in the brain may involve SD.
- “Brain tsunami” has been adopted as a colloquial term for SDs, illustrating their wave-like and deadly nature (https://www.charite-academy.de/). Conversely, whether SDs could be beneficial for survival in the surrounding normal gray matter of mammals has been debated [227]. Such benefits could include upregulation of growth factors, termination of seizure discharge, stress response proteins, and inflammatory mediators. These preconditioning actions could promote plasticity and regeneration and might reduce the vascular steal effect on ischemic zones through the physiological oligemia [3]. Perhaps also, SD is a way that compromised brain tissue signals distress to neighboring regions. Certainly, there are still many fascinating aspects of SD yet to be discovered.
